# Supplementary material for: Social accountability in primary health care facilities in Tanzania: Results from Star Rating Assessment
Source: PLoS One. 2022 Jul 25;17(7):e0268405. doi: 10.1371/journal.pone.0268405 (PMC9312412; doi:10.1371/journal.pone.0268405)
Supplement: Attachment — Submitted filename: ReviewELodenstein.docx [file pone.0268405.s002.docx]

Review manuscript number PONE-D-21-24574

By: E. Lodenstein

03-12-21

**Introduction**

Structure.

- Start with defining what quality is and why it is problematic in TZ
- Then the section on QI
- The social accountability (SA) element of the SRA is the focus of the study. If you decide to mention other forms of accountability as you do on the top of page 5, they need to be explained or you have to leave it out.

I had a difficult time understanding what the SRA is, how it was developed, when it was applied (twice so far, I understand), by whom etc. Is it government initiated? Who defined the 12 domains and the assessment criteria etc. Are these criteria based on government policy (e.g. regarding the functioning of HFGC)? What was the “stepwise improvement process towards an accreditation status”? What is “pre-accreditation”? The information on the SRA is presented at different places (introduction + methods) but you could think about presenting it in one overview- perhaps a box.

Aim of the study (page 6/7)

- The statement of the aim could be reformulated to make it more readable: e.g. This paper aims at describing the status of social accountability in public and private PHC facilities in Tanzania after the three years of implementation of Star Rating Assessment from (2015/16 to 2017/18) as part of the then broad government initiative termed “Big Results Now.
- “Describing the status” is unclear: do you mean “assessing the social accountability performance of PHC”
- “after three years of implementation” suggests you are conducting an assessment and compare results against a baseline situation. However, from the results section I see that you are assessing the performance at one point in time based on data from 2017/2018. In that case, the mentioning of “three years after implementation” is confusing. To simplify, you could just reformulate “….In Tanzania as part of the Star Rating Assessment that was conducted in 2017/2018”.

Specific objectives:

- “during SRA” is unclear. I understand the SRA is just the assessment that takes place at one moment in time, it is not an intervention to improve SA.
- “functional indicators” is unclear. Do you mean “PHC facilities that perform well according to performance indicators of SA”? or “PHC that have functional SA mechanisms in place”?
- The analysis goal mentioned on top of page 7 on performance accountability seems a third specific objective. However, that objective is not achieved in the paper. Also, the concept of “performance accountability” is not explained in the paper, so I suggest to explain it or take it out. Since you position the SA assessment in the context of Quality of Care I would stick to the concept of quality, rather than introducing another concept (performance).

**Methods**

- From the introduction, I understand that there is a standard SRA tool that is being applied in the country across PHCs. I then assume that for the 5 indicators on Social Accountability, the tool already had identified indicators and measurements prior to this study. What is the origin of table 1: is it derived from the standard existing SRA tool (and how was it developed – see comment on introduction before)? OR was it developed by the researchers?
- If it was an existing tool in the context of SRA – how do the literature review and the conceptual framework link to the tool?
- The conceptual framework seems to suggest that 2 out of the 5 indicators are independent variables. However, they are not analyzed as such, can you explain that?

Table 1.

- The title of the table is complex, is it not simply a “tool to assess SA performance”?
- Indicator 1 – functional facility governance committees or boards.
  - Committees can only receive 0 or 6 points (right column). But in the analysis section you state they can also receive 0,5 points. For this indicator, does it mean facilities get 0,5 points if they meet 2,3,4, or 5 characteristics? If so, it should be added in the right column that it is also possible to have a score of 2,3,4,5.
  - Key information on available resources is displayed; the scores do not seem exclusive, e.g. “at least 2” can also be “all three”.

Data collection section: if the data collection, cleaning and compilation is not done by the authors, this section describes not how data were collected for the study, but how the data that make up the national dataset are collected. This is an important difference that needs to be clarified. From my understanding, the authors did not collect the data and did not perform the scoring. They only performed the analysis.

The description of the calculation of the scores seems complicated and can be simplified (page 11). Just explain that a facility could gain 5 points maximum and they needed 4 to be qualified as socially accountable/well performing. A suggestion: “First, the individual scores were calculated (the score for the indicator divided by the maximum possible score x 100 to give a percentage score). Secondly, the total score across the 5 indicators was determined by calculating the average of the percentage scores for the 5 indicators”.

The cut-off point of 80% is described twice on page 12 – this is a repetition.

Data management and analysis (page 12). “the data were checked…” What data are you referring to? The actual data sources (minutes etc..), the scores?

**Results**

- “study participants” seems the wrong term for health facilities. Rather refer to “description of health facilities under study”.
- How do you explain the high number of excluded facilities? What does it say about the methodology or the completeness of the database? (perhaps not discuss in results section but in discussion or study limitations)
- Reference to table 1 should be table 2 in the text
- Page. 13. I think “functional indicators” is not the right formulation as indicators do not function, they are just representations of data. Perhaps something like “proportion of well performing health facilities regarding social accountability” or “proportion of health facilities with functional social accountability practices”.
- Figure 2 title is not correct. What you are presenting is not the proportion but the distribution of performance for different social accountability components. The description of the 5 elements on the left should then also change, leaving out the first part “proportion of facilities that”. You could also make the figure more readable by using words for the legends, e.g. yellow = good performance, orange = average performance, grey = poor performance. In the text (page 13), you state that 45,9% were found to be socially accountable. The average of data in figure 2 however, lead to a percentage of 50,48%. I may be wrong but please check this.
- Page 14. Part of the title “Facility related characteristics…” is not necessary – you can leave out SRA of 2017/2018.
- Page 14. “…achieving social accountability status during assessment…” seems a complicated way of formulating, perhaps alternative: “..had increased likelihood of performing well on social accountability…”

**Discussion**

- Page 15. The statement “…these findings are in line with….” Is very unclear. Performance of what, of whom? What does it mean “cross above the half of the allocated scores”. Which scores? You would need to give some more detail of the study you are referring to.
- The section on health facility governing committees speaks about many other elements that are not related to the 6 sub-indicators assessed under this component (e.g. non-responsiveness was a separate indicator, commitment of health care workers is again another topic, and independence of the committee was not assessed in the SRA..). I suggest you stick to your findings in the discussion section and compare with findings of similar studies.
- The section on display of information: rather than comparing with Kenya, it may be more relevant to discuss why the display of information may have been low. Is it not a requirement from the MoH, do the facilities have this information? And if you do not know, it may be a question for further research.
- The argument on health facilities addressing local concerns: you suggest a link between poor functioning health committees and addressing local concerns but the indicator was about facility management addressing concerns and not facility committees. Please check this argument again. Also, given your conceptual framework, could you have done a statistical analysis of this association between functionality of committees and addressing local concerns?
- Section on engagement of health providers could involve a discussion on whether engagements around public health education and campaigns constitute an element of social accountability. In my view, having meetings with communities around immunization (with evidence in minutes) does not say much about the quality of engagement and whether these meetings are opportunities for communities to call health providers to account. In literature, e.g. McCoy, there is a discussion about the public health/health education versus social accountability role.
- Page 19. This is an unclear statement, please elaborate: “Limited findings are contrary to ours whereby private-owned facilities performed better than public-owned facilities”. Which findings, from whom, about what?
- Maybe you could replace one of the sections above by a discussion on how the SA elements sits within the overall QI initiatives and the overall SRA assessment. So as to refer back to the introduction on QI and the larger SRA process.

**Limitations of the study**

I think you should include limitations on the methods and tools as well. E.g. discuss implications of the following issues for your findings: tool (table 1) :

- No weighing, all indicators evenly important
- Scoring often dependent on one data source: minutes
- Complaints, issues, or concerns are different things, may be treated differently by the boards and facility managers. No distinction made.
- “Facility addressed local concerns”, seems a difficult one to assess as probably hard to link concerns to plans and vice versa. Also, it is communities who should assess this performance rather than external assessors.

**Conclusion**

- The first section seems to present an invalid statement – you did not compare between 2015 and 2017/2018 so you cannot make the statement that the situation improved. Also, your findings do not suggest that SRA can be a mechanism for performance accountability.
- The recommendation is interesting but maybe you can be more specific: training on the use of SRA assessment, or on the actual use of the tool and scores (e.g. displaying them or discussing them with service users to identify improvements etc.) so that it becomes a SA dialogue tool in health facilities?
- Perhaps include a reflection on the need for further research on the quality of SA/feedback process. The assessment scores are a simple representation of reality, it would be interesting to further understand the processes of feedback, and the level of satisfaction service users have with the SA mechanisms etc.

Overall comments:

- Light proof editing of the English is necessary, especially to check lack or incorrect use of articles.
- There is a useful checklist on reporting on social accountability initiatives. Perhaps it can inspire you to complete the description of the initiative etc. <https://www.heardproject.org/social-accountability/sa-resources/sa-resources-reporting-checklist-overview/>
