# Supplementary material for: Social accountability in primary health care facilities in Tanzania: Results from Star Rating Assessment
Source: PLoS One. 2022 Jul 25;17(7):e0268405. doi: 10.1371/journal.pone.0268405 (PMC9312412; doi:10.1371/journal.pone.0268405)
Supplement: Attachment — Submitted filename: Response to reviewers.docx [file pone.0268405.s003.docx]

On behalf of the co-authors, we thank E. Lodestein for her very constructive comments and guidance. This was one of the best Reviewer we have ever met. We also thank you Editor for ensuring we meet right reviewers.

Dear editor, we humbly submit the responses to comments raised by the reviewer. Thank you again for giving us the opportunity.

Review manuscript number PONE-D-21-24574

By: E. Lodenstein

03-12-21

**Introduction**

Structure.

- Start with defining what quality is and why it is problematic in TZ
- Then the section on QI
- The social accountability (SA) element of the SRA is the focus of the study. If you decide to mention other forms of accountability as you do on the top of page 5, they need to be explained or you have to leave it out.

Thank you for the comment to improve the structure of our manuscript. Quality has been defined and explanation on why it is a problem in Tanzania has been added. The other forms of accountability listed on top of page 5 have been deleted. All changes are shown in track changes in the revised manuscript.

I had a difficult time understanding what the SRA is, how it was developed, when it was applied (twice so far, I understand), by whom etc. Is it government initiated? Who defined the 12 domains and the assessment criteria etc. Are these criteria based on government policy (e.g. regarding the functioning of HFGC)? What was the “stepwise improvement process towards an accreditation status”? What is “pre-accreditation”? The information on the SRA is presented at different places (introduction + methods) but you could think about presenting it in one overview- perhaps a box.

Aim of the study (page 6/7)

- The statement of the aim could be reformulated to make it more readable: e.g. This paper aims at describing the status of social accountability in public and private PHC facilities in Tanzania after the three years of implementation of Star Rating Assessment from (2015/16 to 2017/18) as part of the then broad government initiative termed “Big Results Now.

Thank you for your comment. The sentence has been reformulated as you have suggested.

- “Describing the status” is unclear: do you mean “assessing the social accountability performance of PHC”

Thank you for the comment. This has been rephrased to “*assessing the social accountability performance of public and private PHC facilities*”

- “after three years of implementation” suggests you are conducting an assessment and compare results against a baseline situation. However, from the results section I see that you are assessing the performance at one point in time based on data from 2017/2018. In that case, the mentioning of “three years after implementation” is confusing. To simplify, you could just reformulate “….In Tanzania as part of the Star Rating Assessment that was conducted in 2017/2018”.

Thank you for this observation. The sentence has been edited to make it more concise as you suggested.

Specific objectives:

- “during SRA” is unclear. I understand the SRA is just the assessment that takes place at one moment in time, it is not an intervention to improve SA. This has been deleted and hence the first specific objective has been rephrased to read as follows: “To determine proportion of PHC facilities with functional of social accountability mechanisms ***based on the SRA results***.”
- “functional indicators” is unclear. Do you mean “PHC facilities that perform well according to performance indicators of SA”? or “PHC that have functional SA mechanisms in place”? We mean PHC facilities that have functional SA mechanisms in place. This has been edited in the specific objective number 1.
- The analysis goal mentioned on top of page 7 on performance accountability seems a third specific objective. However, that objective is not achieved in the paper. Also, the concept of “performance accountability” is not explained in the paper, so I suggest to explain it or take it out. Since you position the SA assessment in the context of Quality of Care I would stick to the concept of quality, rather than introducing another concept (performance). Thank you for this observation. The SRA had a countrywide target of all PHC facilities to improve to 3-stars and above, *which very much had a nudge effect* to PHC facilities staff to perform better. Therefore, the sentence has been rephrased to reflect this and the “performance accountability has need defined.

**Methods**

- From the introduction, I understand that there is a standard SRA tool that is being applied in the country across PHCs. I then assume that for the 5 indicators on Social Accountability, the tool already had identified indicators and measurements prior to this study. What is the origin of table 1: is it derived from the standard existing SRA tool (and how was it developed – see comment on introduction before)? OR was it developed by the researchers?

Thank you for your comments. Table 1 originated (derived) from the SRA Tool and modified to a language of publication.

- If it was an existing tool in the context of SRA – how do the literature review and the conceptual framework link to the tool? Thank you for this observation. The literature review looked at existing literature based on the indicators of social accountability in the tool. Also, the conceptual framework was linked to the indicators, however its design was a bit disconnected to the way the data analysis was done. This has been corrected. Now all the indicators are independent variables which contribute to functional social accountability mechanisms in a PHC facility.
- The conceptual framework seems to suggest that 2 out of the 5 indicators are independent variables. However, they are not analyzed as such, can you explain that? This has been corrected. Now all the indicators are independent variables which contribute to functional social accountability mechanisms in a PHC facility.

Table 1.

- The title of the table is complex, is it not simply a “tool to assess SA performance”?

The title is now as simple as “A tool to assess Social Accountability performance at healthcare facilities in Tanzania”

- Indicator 1 – functional facility governance committees or boards.
  - Committees can only receive 0 or 6 points (right column). But in the analysis section you state they can also receive 0,5 points. For this indicator, does it mean facilities get 0,5 points if they meet 2,3,4, or 5 characteristics? If so, it should be added in the right column that it is also possible to have a score of 2,3,4,5.

Not all indicators had an option of Partial (0.5) scores. Only indicator number 3 had three options; Yes (1.0), Partial (0.5), and (0.0). The other 4 indicators had either a Yes or No score. We aimed to communicate this message by the sentence “Each question from the checklist had up to three responses: ‘Yes’ (score=1) or ‘No’ (score=0) or ‘Partial’ (score=0.5)”. However, since the sentence was still confusing; we restructured it to *“All questions had two responses Yes’ (score=1) or ‘No’ (score=0) except the question on the “Key information on available resources is displayed” indicator that had the addition of ‘Partial’ (score=0.5)”*

- Key information on available resources is displayed; the scores do not seem exclusive, e.g. “at least 2” can also be “all three”.

Thank you for this observation. We did a typo error when we were re-writing the tool for publication. The correct sentence should have been “Partial=0.5 was given if 2 items displayed “ and not “Partial=0.5 was given if at least 2 items displayed”

Data collection section: if the data collection, cleaning and compilation is not done by the authors, this section describes not how data were collected for the study, but how the data that make up the national dataset are collected. This is an important difference that needs to be clarified. From my understanding, the authors did not collect the data and did not perform the scoring. They only performed the analysis.

Yes, madam, the authors did not collect the data. We have restructured the data collection section to reflect our role as authors in this study and to avoid confusion.

The description of the calculation of the scores seems complicated and can be simplified (page 11). Just explain that a facility could gain 5 points maximum and they needed 4 to be qualified as socially accountable/well performing. A suggestion: “First, the individual scores were calculated (the score for the indicator divided by the maximum possible score x 100 to give a percentage score). Secondly, the total score across the 5 indicators was determined by calculating the average of the percentage scores for the 5 indicators”.

Thank you, madam. All that you suggested have been included in our revised document.

Moreover, after scanning thoroughly on how this outcome was calculated, we observed that there was an error in formulating the equation. We corrected and repeated analyses that are related to this variable. Therefore, please accept the changes that have occurred in the Abstract, Results, Discussion sections.

The cut-off point of 80% is described twice on page 12 – this is a repetition.

Thank you, madam. The repetition is omitted in a revised document.

Data management and analysis (page 12). “the data were checked…” What data are you referring to? The actual data sources (minutes etc..), the scores?

We were referring to data that is kept in the national database. However, we agree with you that the statement was confusing and decided to restructure the section.

**Results**

- “study participants” seems the wrong term for health facilities. Rather refer to “description of health facilities under study”.

Thank you for the comment. We agree with you and adopted the suggestion

- How do you explain the high number of excluded facilities? What does it say about the methodology or the completeness of the database? (perhaps not discuss in results section but in discussion or study limitations)

The discussion about the high number of excluded facilities is now presented in the study limitation sub-section

- Reference to table 1 should be table 2 in the text

Thank you for the observation. The correction has been made.

- Page. 13. I think “functional indicators” is not the right formulation as indicators do not function, they are just representations of data. Perhaps something like “proportion of well performing health facilities regarding social accountability” or “proportion of health facilities with functional social accountability practices”.

We thank you for the comment and agree with you. “functional indicators” has been replaced by “Proportion of health facilities with functional social accountability mechanisms”

- Figure 2 title is not correct. What you are presenting is not the proportion but the distribution of performance for different social accountability components. The description of the 5 elements on the left should then also change, leaving out the first part “proportion of facilities that”. You could also make the figure more readable by using words for the legends, e.g. yellow = good performance, orange = average performance, grey = poor performance. In the text (page 13), you state that 45,9% were found to be socially accountable. The average of data in figure 2 however, lead to a percentage of 50,48%. I may be wrong but please check this.

Thank you, madam, this is another very important observation from you. We agree with you and changes have been made accordingly. However, there are two things to distinguish here..30.4% (previous 45.9%) are facilities that scored 4 out 5 components=socially accountable AND 50.5% is an average score from all five components (The facilities scored half of allocated 100 points for the five indicators)

- Page 14. Part of the title “Facility related characteristics…” is not necessary – you can leave out SRA of 2017/2018.

Thank you for the comment. The part of the title has been omitted.

- Page 14. “…achieving social accountability status during assessment…” seems a complicated way of formulating, perhaps alternative: “..had increased likelihood of performing well on social accountability…”

Thank you for the comment. The sentence has been restructured

**Discussion**

- Page 15. The statement “…these findings are in line with….” Is very unclear. Performance of what, of whom? What does it mean “cross above the half of the allocated scores”. Which scores? You would need to give some more detail of the study you are referring to.

Thank you for the comment. The paragraph has been modified to give a clear message and the details of the studies referred to have been given.

- The section on health facility governing committees speaks about many other elements that are not related to the 6 sub-indicators assessed under this component (e.g. non-responsiveness was a separate indicator, commitment of health care workers is again another topic, and independence of the committee was not assessed in the SRA..). I suggest you stick to your findings in the discussion section and compare with findings of similar studies.

Thank you for the good comment. Now the discussion has been restructured to focus on sub-indicators of health facility governing committees.

- The section on display of information: rather than comparing with Kenya, it may be more relevant to discuss why the display of information may have been low. Is it not a requirement from the MoH, do the facilities have this information? And if you do not know, it may be a question for further research.

Thank you for the comment.

Yes, we were not able to find why few facilities displayed the information by using the database we have. However, we have presented what our colleagues found two years ago regarding the reasons why the display of data is poor among the facilities.

- The argument on health facilities addressing local concerns: you suggest a link between poor functioning health committees and addressing local concerns but the indicator was about facility management addressing concerns and not facility committees. Please check this argument again. Also, given your conceptual framework, could you have done a statistical analysis of this association between functionality of committees and addressing local concerns?

The facility committees have a role to hold the facility management accountable to their performance. Therefore, if a committee does not follow up on this in their meetings, then there may be laxity in the facility management. Nevertheless, it might not be possible to associate the functionality of committees and address local concerns; they are both predictors of social accountability.

Later on, you also advised replacing one of the sections above by a discussion on how the SA elements sits within the overall QI initiatives and the overall SRA assessment. We agreed with you and therefore decided to omit the section “health facilities addressing local concerns”.

- Section on engagement of health providers could involve a discussion on whether engagements around public health education and campaigns constitute an element of social accountability. In my view, having meetings with communities around immunization (with evidence in minutes) does not say much about the quality of engagement and whether these meetings are opportunities for communities to call health providers to account. In literature, e.g. McCoy, there is a discussion about the public health/health education versus social accountability role.

The SRA tool assessed whether there were minutes at the community authority’s office that show the participation of health providers at the community level. This could be one of the limitations of the tool since the quality of the meeting was not clearly assessed. We have pointed out this weakness in the manuscript text.

The section has been modified to suit the suggestions you provided.

- Page 19. This is an unclear statement, please elaborate: “Limited findings are contrary to ours whereby private-owned facilities performed better than public-owned facilities”. Which findings, from whom, about what?

Clearer details about the studies cited in the text have been given. They were all about social accountability

- Maybe you could replace one of the sections above by a discussion on how the SA elements sits within the overall QI initiatives and the overall SRA assessment. So as to refer back to the introduction on QI and the larger SRA process.

The section regarding “health facilities addressing local concerns” has been omitted to give a space for the section you suggested.

The role of SA elements and SRA in relationship to Tanzanian QI initiatives has been described under a section titled “The role of SA assessment in Tanzanian QI initiatives”

**Limitations of the study**

I think you should include limitations on the methods and tools as well. E.g. discuss implications of the following issues for your findings: tool (table 1) :

- No weighing, all indicators evenly important
- Scoring often dependent on one data source: minutes
- Complaints, issues, or concerns are different things, may be treated differently by the boards and facility managers. No distinction made.
- “Facility addressed local concerns”, seems a difficult one to assess as probably hard to link concerns to plans and vice versa. Also, it is communities who should assess this performance rather than external assessors.

Thank you for these great inputs. We agree with you and we have included them based on the context.

**Conclusion**

- The first section seems to present an invalid statement – you did not compare between 2015 and 2017/2018 so you cannot make the statement that the situation improved. Also, your findings do not suggest that SRA can be a mechanism for performance accountability.

The confusing phrases “improved situation” and “mechanism for performance accountability” have been omitted.

- The recommendation is interesting but maybe you can be more specific: training on the use of SRA assessment, or on the actual use of the tool and scores (e.g. displaying them or discussing them with service users to identify improvements etc.) so that it becomes a SA dialogue tool in health facilities?

Your suggestion has been accepted and changes have been made.

- Perhaps include a reflection on the need for further research on the quality of SA/feedback process. The assessment scores are a simple representation of reality, it would be interesting to further understand the processes of feedback, and the level of satisfaction service users have with the SA mechanisms etc.

Thank you for the reminder. An important reflection on the need for further research has been added.
