# Supplementary material for: Social accountability in primary health care facilities in Tanzania: Results from Star Rating Assessment
Source: PLoS One. 2022 Jul 25;17(7):e0268405. doi: 10.1371/journal.pone.0268405 (PMC9312412; doi:10.1371/journal.pone.0268405)
Supplement: Attachment — Submitted filename: Response to reviewers.docx [file pone.0268405.s004.docx]

Dear editor, we humbly submit the responses to comments raised by the reviewers including you. Thank you again for giving us the opportunity.

Additional Editor Comments:

This report has improved since the last version. However, there are still some more outstanding issues, particularly those raised by one of the reviewers below.

1. The main outcome of the analysis is an ordinal score of accountability congregating different dimensions (collected as a binary variable). The authors continue the classic approach of dichotomization of such kind of score. It is OK but that has the effect of throwing away the ordinal information among those below the cut-off.

Dear Editor, we agree with you that this is one of the great challenges for binary logistic regression. Thank you for the reminder.

2. Please do not just report p-values and the point estimate of odds-ratio (OR) only. Please report the 95% confidence interval of OR.

Thank you for your comment. The 95%CI of OR are now included.

3. Table 3:
- Call it multivariable (not multivariate)

The change has been adapted.

- Add below the table (as a footnote) what variables were used to adjust for.

We have added the footnote explaining which variables have been adjusted for.

4. Stata is not an acronym. Please write Stata not STATA. And please add a citation.

The change has been done and a citation is provided.

**Reviewers' comments:

Reviewer's Responses to Questions**

Reviewer #1: Review of revision 1
Elsbet Lodenstein
14 feb. 2022

The authors have made very important revisions. Comments have been adequately addressed. The clarifications and changes in the calculation make the study more consistent and trustworthy and the discussion is relevant and interesting.

Thank you for the compliment

Revision needed in Methods section:
- The authors need to explain how table 1 was constructed just as they do in the responses to the reviewer. So, basis is SRA tool but complemented with criteria from literature. It needs to be described explicitly which elements are from the original SRA tool and which elements were added by the authors.

The statement on the originality of Table 1 has been added to the manuscript. Table.1 was derived from the SRA Tool and modified to a language of publication, however, none of the indicators was changed.
- In the responses to reviewer, the authors state that “Now all the indicators are independent variables which contribute to functional social accountability mechanisms in a PHC facility”. However, in the text, under study variables this was not yet adapted.

Thank you for noting this dear Reviewer. The sentences have been modified to adapt to the changes.

Other minor revisions:
- Page 3 bottom – Big Results Now initiative needs to be referenced.

The reference has been added.
- Top page 5: definitions of SA need a reference.

The reference has been added.

- Page 5, listing of 5 elements assessed (also mention that it is five elements) – formulation can be simplified and made into nouns. E.g. healthcare workers engagement with the local community; facility addressing local concerns; community participation in facility planning process etc…

The phrase “five indicators” has been added. The formulation had been made to nouns as suggested.
- The “Hence….” sentence could be taken out as objective of the paper is well explained below on page 7.

The sentence has been taken out.

- Page 7. Aim statement improved but I think it is still complicated. ….”SRA Tools to assist as a mechanism for making facility in-charges and other staff accountable in ensuring good performance of their facility in terms of providing quality services”. Why not just …”SRA Tools to assist as a mechanism for making facility in-charges and other staff accountable for providing quality services”. And leave the performance out. I would also exclude the concept of performance accountability because it is again confusing as the paper focuses simply on performance of social accountability, not performance in terms of agreed targets.

Your suggestion on simplifying the sentence has been adopted and the concept of performance accountability has been excluded as well to avoid confusion.

Results
- the sentence on page 14 “The average score in percentages….” should be explained more clearly. Something like “This means that facilities’ overall score for performance of social accountability across the 5 indicators was 50,5%.
Thank you, your suggestion has been adopted in manuscript text.

I recommend to have an English editor conduct proof reading. Overall the text is well written, but the proofreading would take out some omissions, like
- missing articles (e.g. “a” is missing before “variety” on top of page 4; “the” is missing before “development on page 6 or “The” is missing before “Composition” on page 6; there are many similar small mistakes).

Thank you, I have worked on it.

- Formulations: e.g. p. 12 sentence does not run smoothly. “Missing data were not imputed and therefore excluded the in analysis”.

The sentence has been reconstructed.

**Reviewer #2:** The authors have revised a paper describing the results of the Social Accountability measures from the 2018=2019 survey done part of the Tanzania Star Rating Assessment. The paper is important in highlighting the importance o this component of facility performance and they should be applauded for the work. They have also worked hard to respond to another reviewers comments, which have improved the readability and strength of the paper. I however still struggled with a number of areas and some of the writing needs to be reviewed. In particular, while they re clear in their response that this is a single cross sectional report in their responses, there are still areas where the language implies change over time (“how star rating improved Social accountability \” in the introduction and describing how areas were affected when they are only associated in the discussion.

Thank you for the constructive comment and compliment. In any case, when the word “improved” is mentioned throughout the document, it is for the purpose of showing the situation has changed when compared to findings from previous studies. The references are cited in the text. We did not compare the baseline and re-assessment findings.

Introduction
While there is an interesting introduction, I got a bit lost in the discussion about accountability (is it the country which is held accountable to existing and emerging social concerns or in this area-the facility and health system?
We got the reviewer’s concern. We have improved the confusing sentence from *“In the context of PHC, social accountability is a measure of whether a country is held accountable to existing and emerging social concerns and priorities based on need” to “In the context of PHC, social accountability is a measure of whether a country and especially the health facility, are held accountable to existing and emerging social concerns and priorities based on need”*

In the description of how the HFGC is formed, what does “transparent manner at respective levels” mean?
“transparent manner at respective levels” means the members were publicly selected to represent the community at the level of their authorities i.e. Hospital Advisory Body members would represent a community that is served by council hospital (about 250,000 population) while members of HFGC at the Dispensary level would represent the community at specific catchment area of about 10,000 population. The sentence has been rephrased to be more understandable.

I was still confused about the introduction of a conceptual model on page 6 (Molyneux) and Lodensteins definition of social accountability. Is this about the reasons why these structures are valid? And so why they should be a measure of social accountability?

The definitions by these experts have just happened to be in-line with how Tanzania conceptualized on indicators needed to assess social accountability at primary health facilities. However, as explained in limitation section of the manuscript text; we expect the SRA tool will be modified in next days to incorporate views from more scholars and practioners.

I am also a bit confused about the aims-particularly “the analysis aims at showing the potential of the SRA tools to assist as a mechanism for making facility in-charges and other staff accountable….” I did not see that in results or discussion?

Thank you for the good comment. Since the SRA tool included an assessment on whether the facility in-charges and other staff were socially accountable; the findings from the assessment will highlight the areas that need improvement from which facilities/ministries can work upon. The sentence has been modified to make it clearer.

Methods
Table 1 I assume is not just a tool, but actual the section from the STAR assessment? Consider adding that

Your comment is well adapted. We have changed the title from “A tool assess Social Accountability Performance at Healthcare Facilities in Tanzania” to “A section on SRA tool assessing Social Accountability Performance at Healthcare Facilities in Tanzania”

Why is a 2 point assessment discussed when only one assessment is used (would be very interesting to see change over time)

Referring to the above responses, we did not compare the baseline and re-assessment findings and therefore whenever the word improved used; it was compared to past findings in the country.

How were the independent factors chosen? Were there others (such s overall Star rating?)

We could add more variables, however, we were limited to very few which are in the current database. This has been included in the limitations of the study.

The use of the term performance scores is a little confusing-do you mean the SA scores?

The sentence has been modified to avoid confusion.

Results:
• I think there is a missing space in the first subtitle “understudy:?

Thank you for the suggestion. However, to the best of our understanding and if you agree with us-we thought it should remain as “understudy” and not “under study”. Or else, because the word itself brings some confusion.. we have opted to change the title to “Description of participating health facilities”

• Importantly-why did 58.4% not meet eligibility-would consider a consort diagram f they fell out by different reasons. This is also a major potential bias?

Yes, this was one of the major limitations of the study. We excluded a high number of facilities that did not meet our exclusion criteria and this could relatively affect the strength of our study. Nevertheless, this is the first Tanzanian study on social accountability assessment having National coverage of PHC facilities. The facilities were randomly excluded (not systematic error) and therefore less chance to cause selection bias

• It would be helpful to also see the distribution of the results which make up the Functional facility governance committee or boards. What were the areas which resulted in 0? Even a description of the results from 0-6 would be of great interest. Some seem a bit subjective “adequately trained”.

We agree with you. However, this was not in the scope of study provided the dataset prepared. We look forward to meeting your wish which is also ours. Thank you.

• Can the authors include the actual tool and any scoring information in terms of how the data were collected. If there were no minutes from committees in the last 6 months-how was that scored as an example but applies to others as depended on documentation

The data collection tool for social accountability area is attached as Table 1. The scoring was done at the indicator level. The facility needed to get all 6 verification questions right, for example; to score YES for the indicator “Functional facility governance committees or boards”.

• In the figure-how can you have overall ratings when ratings were missing from some of the components of the 5 areas? Also the figure (on my print out) seems a bit blurry

The overall ratings were the facilities’ average scores that had no missing values. We tried to improve the clarity in this version.

Discussion

As noted above, you do not know if the determinants affected the rating-more association
The second paragraph is a bit repetitive and describe the methods and results. It would be better to dive into the discussion of the results

Thank you for the comment. The second paragraph has been omitted in this version.

There is a sentence alone “experience has shown the existence of poor social accountability mechanisms among health facilities in Tanzania-whose experience and needs expansion (and references. How does this support or not your findings.

The sentence has been modified to suit the reviewer’s recommendation.

For the part of the HFGC-which components gave the most challenge?

It was impossible to discuss at the question level because the data were collected and analysed at the indicator level.

Are there any references showing the impact of well run committees?. The statement about HFGCs being ineffective also needs a bit more detail and again if and how similar to or different from results

Some sentences and references have been added to show evidence of some impact of well-run committees and also to detail infective HFGCs

The statement about use of records to score (which I think is important) however may belong better in limitations unless you are discussing the instrument itself (versus study)

Thank you for this observation. The paragraph has been improved and thereafter transferred to Limitation section.

The section on displayed information is interesting, however the discussion about data analysis does not seem to be as relevant as the displayed information was budgets and resources?

I agree with you, however, information on “plans and budget, allocation to medicines and supplies, revenue collection, received funds, and expenditure” sometimes would require analysis of data first to get the information to display on the walls. E.g. budget summary, funds and expenditures would require arithmetic calculations

Health work engagement: The authors are correct that the way the indicators was assessed “could have exaggerated the findings”-but wonder if that is true for most or all of the areas measured?

We agree with you that this could be true for other indicators and not for “Health workers’ engagement with the local community” only. Therefore, we have modified the paragraph to reflect what we wanted to communicate. We needed readers to understand that high performance in community engagement could be associated with the country’s high achievement in community healthcare outreach services.

The use of the term “forced” in convening needs explanation-how were they forced?

The more appropriate phrase has been applied in place of the “forced”

Engaging community: The statement about hampering by gaps in manpower, finance and infrastructure is important and could use more detail in terms of what is needed to address the gaps as measured by the STAR SA tool

The details have been added in manuscript text

The section on the role of SRA n improving social accountability was a bit hard to understand as no information as in the results about how these results were used. I think potentially one of the most important potential new insights and would be helpful to know progress particularly now that we are in 2022

In this section, we thought it was important to let the reader understand how the SA elements sit within the overall QI initiatives and the overall SRA assessment. So as to refer back to the introduction on QI and the larger SRA process. This was also the recommendation from one of the reviewers

The authors have done a through job around limitations (see however comment above). However the important statement about updating the SAR is not a part of limitations but perhaps goes netter into a discussion about the tool? Or conclusions and next steps?

Thank you for your comments which we have also included in this new edition

As noted above-the authors need to describe the exclusion criteria (and I think that is a typo as they may have meant ‘did not meet our inclusion criteria)

If I got you right, then we have included the exclusion criteria under the methodology part of the manuscript.

Writing
• The authors have some extremely long sentences which make it hard to a reader to understand the main points. As examples, the end of the very long paragraph in the introduction starting with “to achieve”

We have restructured long sentences but retained the message. Thank you for the comment.

• All acronyms need spelling out (like the first time SRA is used) and would decrease use of ones which are not common (ex HFGC/HFB).

Thank you for the comment. We have cross checked the acronyms and worked on them.

• All quotes should be identified for their source-this is missing in a number of places.

We have added some references to quotes that had not indicated the sources. There was a sentence in the data extraction and management section in which we incorrectly marked quotation; We have removed its quotations.
